# Benign Mesenchymal Tumor of the Vulva: A Case Report and Narrative Review of the Literature

**DOI:** 10.7759/cureus.87807

**Published:** 2025-07-13

**Authors:** George Mpourazanis, Kostas Tepelenis, Maria Kouvara-Pritsouli, Ioannis Adamopoulos, Konstantina Diamadi, Katerina Ntaflou, Christos Akrivis, Nikolaos Tepelenis, Panagiotis Tsirkas, Ruediger Schulz-Wendtland, Minas Paschopoulos

**Affiliations:** 1 Department of Obstetrics and Gynecology, General Hospital of Ioannina G. Hatzikosta, Ioannina, GRC; 2 Department of Surgery, General Hospital of Ioannina G. Hatzikosta, Ioannina, GRC; 3 Faculty of Medicine, University of Ioannina, Ioannina, GRC; 4 School of Social Sciences, Public Health and Policies, Hellenic Open University, Patras, GRC; 5 Department of Environmental Hygiene and Sanitarian Public Health Inspections, Hellenic Republic, Region of Attica, Athens, GRC; 6 Department of Public Health Policy, Sector of Occupational and Environmental Health, School of Public Health, University of West Attica, Athens, GRC; 7 Department of Early Childhood Education, Laboratory of Social Sciences and Education, University of Ioannina, Ioannina, GRC; 8 Department of Anesthesiology, General Hospital of Ioannina G. Hatzikosta, Ioannina, GRC; 9 Department of Internal Medicine, General Hospital of Athens G. Gennimatas, Athens, GRC; 10 Department of Obstetrics and Gynecology, University Breast Center for Franconia, University Hospital Erlangen, Friedrich-Alexander University of Erlangen-Nuremberg, Erlangen, DEU; 11 Department of Obstetrics and Gynecology, University Hospital of Ioannina, Ioannina, GRC

**Keywords:** acrochordon, benign malignancy of the vulva, benign mesenchymal lesion, benign neoplasm of the vulva, fibroepithelial stromal polyp

## Abstract

Acrochordons are benign skin neoplasms that originate from mesenchymal and ectodermal tissues. These lesions are commonly found in regions of the skin that experience friction or folding. This report presents a 48-year-old female patient who exhibited a 3 cm acrochordon located on the right labia majora of her vulva. A thorough review of her medical history did not reveal any identifiable risk factors that could account for the onset and rapid growth of the polyp. A wide surgical excision was conducted, and the subsequent histopathological examination confirmed a polypoid proliferation of the stroma, accompanied by a hypocellular stroma composed of spindle cells and loose collagenous stroma. At the six-month follow-up, the patient showed no signs of recurrence.

## Introduction

Acrochordons, as documented in scientific literature, are commonly recognized as skin tags, soft fibromas, or fibroepithelial polyps (FEPs). These benign growths originate from connective tissue and epidermal layers, having mesenchymal and ectodermal antecedents. The occurrence of these non-cancerous growths in the genital region has been reported in the medical literature since the 1960s [1].

The incidence of these tumors within the global population is reported to be 25%, with their occurrence becoming increasingly prevalent with advancing age [2]. Acrochordons are predominantly observed in women of reproductive age and are typically located in the genital tract, encompassing the vagina, cervix, and vulva. A smaller percentage may also be located in areas characterized by skin folds, such as the neck, axilla, submandibular region, or inguinal region [3,4]. Additionally, the ureter, colon, and chest wall are among the other uncommon anatomical locations where these neoplasms have been reported [5].

The specific cause of these neoplasms is yet unknown based on their genesis. It is posited that hormonal factors or chronic inflammation may potentially initiate the development of the benign neoplasm. It is acknowledged that these tumors are hormone-sensitive, as evidenced by the presence of estrogen and progesterone receptors in the stromal cells of the polyp [6].

We report the case of a 48-year-old woman with a 3 cm acrochordon on the right labia majora of her vulva. Wide surgical excision was the preferred treatment. This study emphasizes the clinical and diagnostic methodologies employed for the evaluation of these lesions. It represents a rare instance of a tumor developing without any identified risk factors. That is why this case is worth describing.

## Case presentation

**Figure 1:**
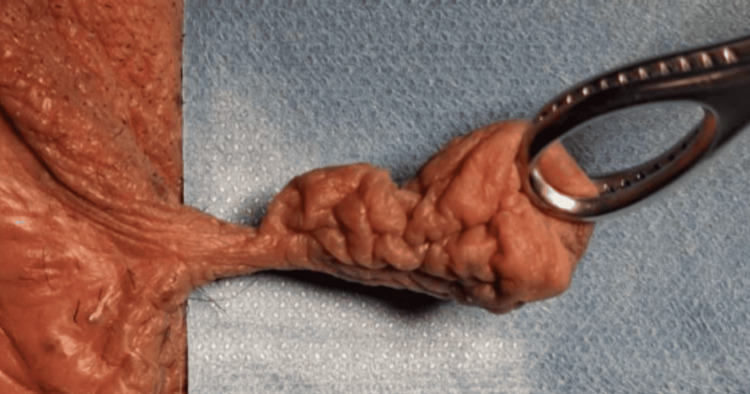
Macroscopic appearance of the acrochordon

A 48-year-old female patient of reproductive age presented to the outpatient gynecologic unit. Her visit was prompted by the presence of a small tumor on her vulva (Figure 1). She reported that the tumor was neither painful nor tender, although she did experience mild discomfort in her external genital area. The vulvar region showed no history of trauma or friction-related injury, and no signs of infection or inflammation were found.

During the clinical examination, a non-painful pedunculated tumor was observed in the genital area, measuring a maximum diameter of 3 cm and extending from the right labia majora. The lesion was palpable with density and was covered with normal skin, without any inflammatory lesions. The patient reported that this mass first manifested one year prior, with significant growth observed in the last four months. She denied experiencing vaginal pain or discharge. The patient deferred seeking medical assistance due to feelings of embarrassment regarding the mass. The woman's menstrual cycle was normal, with typical hormonal status for a premenopausal woman, and no abnormal Pap smear test results were observed. Her body mass index (BMI) was recorded at 23 kg/m². The patient reported no habits of alcohol consumption or smoking.

Her surgical history includes three cesarean sections, a dilation and curettage, a hysteroscopy, and a cervical biopsy. The transvaginal ultrasound revealed a normal uterus with functional adnexa and no fluid in the pouch of Douglas; her laboratory results are listed in Table 1.

**Table 1:** Preoperative and postoperative laboratory results WBC: white blood cell; LYMPH: lymphocyte; HGB: hemoglobin; HCT: hematocrit; INR: international normalized ratio; aPTT: activated partial thromboplastin time; PLT: platelet; CRP: C-reactive protein; AST: aspartate transferase; ALT: alanine transaminase; GGT: gamma-glutamyl transferase; ALP: alkaline phosphatase; ALB: albumin; GLC: glucose; TPR: total protein; UA: uric acid; URE: urea; CRE: creatinine; K+: potassium; Na+: sodium; CA 125: cancer antigen 125; CA 15-3: cancer antigen 15-3; CA 19-9: cancer antigen 19-9; CEA: carcinoembryonic antigen; HCG urine: human chorionic gonadotropin urine

Parameter	Day 0 (admission and operation)	Day 1	Day 3 (exit day)	6-month follow-up	Reference values
WBC	6.76 k/μL	13.5 k/μL	8 k/μL	7 k/μL	4-11 k/μL
Neutrophils	66.8%	78%	55%	45%	40-75%
LYMPH	25.1%	24%	22%	27%	20-45%
HGB	15.3 g/dl	10 g/dl	11.4 g/dl	12.5 g/dl	11.8-17.8 g/dl
HCT	44.7%	33%	38%	40%	36-52%
INR	0.96	0.88	Not taken	Not taken	0.8-1.2
aPTT	27.54 seconds	22.40 seconds	Not taken	Not taken	26-36 seconds
PLT	272 k/μL	275 k/μL	286 k/μL	289 k/μL	140-450 k/μL
CRP	3 mg/dl	2.5 mg/dl	0.25 mg/dl	0.20 mg/dl	0-0.80 mg/dl
AST	16 U/L	20 U/L	44 U/L	24 U/L	5-33 U/L
ALT	29 IU/L	21 IU/L	55 IU/L	29 IU/L	5-32 IU/L
GGT	35 IU/L	15 IU/L	66 IU/L	30 IU/L	5-31 IU/L
ALP	50 IU/L	53 IU/L	59 IU/L	45 IU/L	35-125 IU/L
ALB	3.8 g/dl	3.5 g/dl	4.2 g/dl	3.8 g/dl	3.5-5.1 g/dl
GLC	74 mg/dl	70 mg/dl	90 mg/dl	110 mg/dl	70-115 mg/dl
TPR	6.5 g/dl	6.3 g/dl	6.8 g/dl	8 g/dl	6.2-8.4 g/dl
UA	3.6 mg/dl	2.5 mg/dl	2 mg/dl	4.6 mg/dl	2.3-6.1 mg/dl
URE	17 mg/dl	15 mg/dl	25 mg/dl	44 mg/dl	10-50 mg/dl
CRE	0.71 mg/dl	0.89 mg/dl	0.90 mg/dl	0.98 mg/dl	0.5-1.1 mg/dl
K+	3.8 mmol/l	3.5 mmol/l	3.8 mmol/l	5 mmol/l	3.5-5.1 mmol/l
Na+	138 mmol/l	140 mmol/l	134 mmol/l	140 mmol/l	136-146 mmol/l
CEA	2 ng/ml	-	-	-	<5 ng/ml
CA 125	10 U/ml	-	-	-	<35 U/ml
CA 15-3	15 U/ml	-	-	-	<31.3 U/ml
CA 19-9	18 U/ml	-	-	-	<37 U/ml
HCG urine	Negative	-	-	-	Positive/negative

The clinical examination found no enlarged pelvic lymph nodes. Based on clinical findings, an acrochordon or fibroepithelial tumor was suspected, and the patient was scheduled for surgery. In the operating theater, the patient received general anesthesia and was placed in a lithotomy position. A comprehensive excision of the pedunculated tumor was conducted. The incision site was subsequently closed using a single subcutaneous suture, and the remaining aspects of the operative course were documented unremarkable.

**Figure 2:**
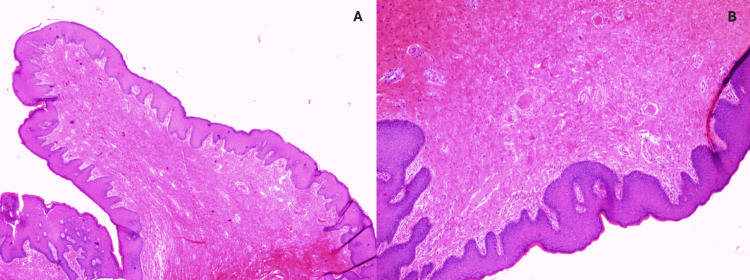
(A) Polypoid proliferation of the stroma with normal overlying squamous epithelium (fibroepithelial polyp). (B) Hypocellular stroma composed of spindle cells and loose collagenous stroma Histological section stained with hematoxylin and eosin

The surgical specimen was forwarded to the pathology department, which exhibited a macroscopically whitish complexion and a fibroelastic consistency, along with a polypoid formation measuring a maximum diameter of 3 cm. The lesion was excised with a skin base, ensuring a tumor margin of 0.5 cm and ''no atypia or mitotic figures'' found. Microscopically, a hematoxylin and eosin (H&E) stain demonstrated a well-defined, hypocellular stroma comprising spindle cells and loose collagenous stroma (Figure 2).

On the third postoperative day, the patient was released from the clinic. There was no relapse of the acrochordon at the excision area during the six-month follow-up period.

## Discussion

Fibroepithelial stromal polyps represent benign mesenchymal tumors that are typically located in the neck, axilla, submandibular region, and inguinal region; however, they may also manifest in the genital area [1,3]. FEPs can be classified into three clinical types: small furrowed papules, usually measuring approximately 2 millimeters in both width and height; filiform lesions, which are around 2 millimeters wide and 5 millimeters tall; and larger, pouch-like protrusions. The term "acrochordon" refers to the smaller lesions, whereas "FEP" is typically utilized to describe the larger ones [7].

The issues surrounding vulvar malignancies, including vulvar cancer, polyps, and acrochordons, highlight significant public health concerns, particularly regarding women's hesitation or reluctance to seek gynecological care due to feelings of shame and embarrassment [8]. This fact emphasizes the critical need for public awareness campaigns aimed at destigmatizing the vulva. Such actions could empower women to confront their fears and seek medical attention earlier, potentially leading to improved healthcare and better outcomes [8,9].

The psychological, relational, and sexual difficulties that arise as a consequence of this diagnosis highlight the significant impact on women's mental health and intimate relationships [8-10]. This perspective underscores the importance of addressing societal perceptions that contribute to delays in seeking medical expert opinion and late presentations of this disease [10,11].

There is a critical intersection between public health awareness and the psychosocial aspects of vulva malignancies [12]. Research suggests that enhancing awareness and providing comprehensive support can lead to improved health-seeking behaviors among women, ultimately addressing the stigma and psychological burdens associated with these conditions [13,14].

These tumors can have a psychological impact on affected women. Research has shown that when a woman's medical history includes a mass concerning gynecological cancer, this fact is linked to a higher risk of developing depression, anxiety, and adjustment disorders [15]. The intersection of public health awareness and the psychosocial aspects of vulvar malignancies highlights that enhancing awareness and providing holistic support can significantly improve health-seeking behaviors among women. Addressing the stigma and psychological burdens associated with these conditions is crucial for fostering a supportive environment that encourages timely medical intervention and enhances overall health outcomes [16]. The importance of healthcare professionals engaging in proactive communication and education is emphasized, as this can play a pivotal role in changing societal perceptions and improving health-seeking behaviors among women [17]. The clinical results and psychological effects of medical conditions of the vulva are presented in Table 2.

**Table 2:** Reported relevant bibliography on vulva pathology with results and psychological effects

Author/year	Type of study	Number of patients	Age	Vulva pathology	Psychological issue	Results
Iraola et al. 2024 [8]	Original research article	25	25-52	Not mentioned	Embarrassment and discomfort	Physical and psychological violence
Cambaz Ulaş et al. 2025 [9]	Qualitative study	90	37.29 ± 10.14	Not mentioned	Ache/pain for the gynecological examination	Metaphors may reflect physical and emotional experience during examination
Malandrone et al. 2021 [10]	Literature review	30	56.3	Vulvar cancer	Women with vulvar cancer have depressive and anxiety symptoms	Care providers should implement an integrated care model to help women with vulvar cancer recognize and address their unmet needs
Bradfield Strydom et al. 2022 [11]	Research article	10	45.4	Recurrent vulvovaginal candidiasis	Recurrent vulvovaginal candidiasis significantly impacts women physically and psychologically	Affected women with recurrent vulvovaginal candidiasis have emotional and psychological issues
Zach et al. 2024 [12]	Original research article	105	-	Vulvar cancer	Anxiety levels among women decreased from 42% to 30% within 12 months, with factors like insomnia and information needs contributing to higher anxiety levels	Newly diagnosed vulvar cancer patients often experience high anxiety levels, but improvement is evident during follow-up, suggesting that targeted interventions targeting insomnia, vulvar symptoms, and unmet needs may reduce anxiety
Olesen et al. 2023 [13]	Review	70	44.3	Vulvar cancer	Women felt alone and had their needs unmet	Healthcare professionals require training to break taboos and address the sexual health needs of vulvar cancer patients. This includes doing systematic screenings from a multifaceted approach
Sousa Rodrigues Guedes et al. 2022 [14]	Systematic review	16	25-69	Gynecological cancer	Women with cervical and breast cancer have a higher risk of developing sexual dysfunction	Cancer treatments increase the risk of sexual dysfunction in women
Korkontzelos et al. 2023 [15]	Case report	1	45	Fibroepithelial stromal polyp	Bipolar disorder, adjustment disorder, and anxiety	Surgical excision of the benign neoplasm
Bentham et al. 2021 [16]	Research article	202	Not mentioned	Vulval lichen sclerosus	Some women have experienced relationship collapse due to the condition, and many feel ashamed of their symptoms	Teaching programs can help women in seeking medical advices for conditions like vulval lichen sclerosus
Hosseini et al. 2021 [17]	Research article	150	Not mentioned	Genital warts	The health belief model can enhance women's preventive behaviors and psychological effects of genital warts, and expert education should be provided regularly in healthcare centers	There were no significant differences in awareness, perceived susceptibility, severity, benefits, barriers, and self-efficacy after an educational intervention

In our study, the histopathology report showed no signs of malignancy, and there was no evidence of recurrence after a six-month follow-up. More studies should be conducted soon to explore the development and growth mechanisms of these tumors in detail and gain a deeper understanding of their exact nature.

## Conclusions

This case emphasizes the necessity of increased clinical awareness of such uncommon lesions with no any risk factors already known according to the scientific literature by highlighting a rare appearance of an acrochordon in the vaginal region. Despite being benign, patients may experience severe anxiety and shyness due to their unique placement and presentation. This fact may put the patients off seeking early and timely health professional examination and subsequent medical attention and treatment. Differentiating these polyps from other possibly cancerous growths requires an accurate diagnosis backed by histological analysis. In addition to ensuring positive clinical outcomes, early detection and prompt surgical management also enhance patient comfort and mental health. Clinicians should consider benign FEPs in the differential diagnosis of vulvar masses.

Future research with comparable cases will be crucial to further our understanding of the pathophysiology of these lesions, direct adequate management options, and educate best practices in patient care, as their origin and behavior are still poorly understood.
